# Using a Facebook Forum to Cope With Narcolepsy After Pandemrix Vaccination: Infodemiology Study

**DOI:** 10.2196/11419

**Published:** 2019-04-16

**Authors:** Karin Blomberg, Mats Eriksson, Rickard Böö, Åke Grönlund

**Affiliations:** 1 School of Health Sciences Örebro University Örebro Sweden; 2 School of Business Örebro University Örebro Sweden

**Keywords:** narcolepsy, mass vaccination, social media

## Abstract

**Background:**

In 2010, newly diagnosed narcolepsy cases among children and adolescents were seen in several European countries as a consequence of comprehensive national vaccination campaigns with Pandemrix against H1N1 influenza. Since then, a large number of people have had to live with narcolepsy and its consequences in daily life, such as effects on school life, social relationships, and activities. Initially, the adverse effects were not well understood and there was uncertainty about whether there would be any financial compensation. The situation remained unresolved until 2016, and during these years affected people sought various ways to join forces to handle the many issues involved, including setting up a social media forum.

**Objective:**

Our aim was to examine how information was shared, and how opinions and beliefs about narcolepsy as a consequence of Pandemrix vaccination were formed through discussions on social media.

**Methods:**

We used quantitative and qualitative methods to investigate a series of messages posted in a social media forum for people affected by narcolepsy after vaccination.

**Results:**

Group activity was high throughout the years 2010 to 2016, with peaks corresponding to major narcolepsy-related events, such as the appearance of the first cases in 2010, the first payment of compensation in 2011, and passage of a law on compensation in July 2016. Unusually, most (462/774, 59.7%) of the group took part in discussions and only 312 of 774 (40.3%) were lurkers (compared with the usual 90% rule of thumb for participation in an online community). The conversation in the group was largely factual and had a civil tone, even though there was a long struggle for the link between the vaccine and narcolepsy to be acknowledged and regarding the compensation issue. Radical, nonscientific views, such as those expounded by the antivaccination movement, did not shape the discussions in the group but were being actively expressed elsewhere on the internet. At the outset of the pandemic, there were 18 active Swedish discussion groups on the topic, but most dissolved quickly and only one Facebook group remained active throughout the period.

**Conclusions:**

The group studied is a good example of social media use for self-help through a difficult situation among people affected by illness and disease. This shows that social media do not by themselves induce trench warfare but, given a good group composition, can provide a necessary forum for managing an emergency situation where health care and government have failed or are mistrusted, and patients have to organize themselves so as to cope.

## Introduction

### Background

In 2010, newly diagnosed narcolepsy cases among children and adolescents were reported in several European countries as a consequence of comprehensive national vaccination campaigns with Pandemrix against H1N1 influenza (swine flu) that took place during the winter of 2009–2010 [1]. In Sweden, approximately 350 young persons acquired narcolepsy after vaccination against swine flu [2,3]. Narcolepsy is a neurological disease that involves a disturbed regulation of wakefulness and sleep. The main symptoms are severe insomnia and daytime sleep attacks that cannot be controlled. Cataplexy, a sudden loss of muscle tone and a feeling of paralysis, is commonly associated with narcolepsy and can have a significant impact on the daily lives of the affected persons. There is no curative treatment, only symptom relief with stimulant drugs, antidepressants, and sodium oxybate (Xyrem), which all have fairly severe adverse effects [4].

Despite a lack of scientific documentation of the life situations of young persons who acquired narcolepsy after swine flu vaccination, several personal descriptions have been published in the media (newspapers and the internet) and aired on television. Having grown up in the digital age, young people today are accustomed to expressing their opinions quickly and widely on social media. In a crisis, social media could potentially be a significant means for disseminating and collecting information and could improve emergency management, for example. On the other hand, social media have been ascribed the power to misinform [5]. Social media are described as an amplifier of opinions more than as an arena for objective discussions [6-8], and the health information presented is often inaccurate or not in line with official recommendations for prevention and treatment. For many people, attitudes toward vaccination are shaped not just by health care but also by other information sources published on websites and social media. In the case of vaccination campaigns, several antivaccination movements opposed to, for example, vaccination against human papillomavirus and measles have been started through social media [9,10]. The relation between narcolepsy and the swine flu vaccination campaign is often used in blogs as an example of why the medical authorities should not be trusted [11].

As social media and the internet are seen by many as a primary source of health-related information [12,13] and can change how young people share information and make decisions regarding their health and well-being, it is important to investigate how information, perceptions, and attitudes are spread. The power of social media puts new demands on communication strategies of health care systems.

The swine flu virus was first isolated from pigs in 1931. The first known death of a human due to the virus was described in 1976. In 2009, a new type of swine flu virus emerged in California, USA, which was quite harmless to most people, whereas some individuals who did not belong to traditional risk groups became extremely ill. Within a few months, the flu became pandemic, and many countries decided to offer their citizens a pandemic vaccine campaign [1]. In connection to the 2009–2010 pandemic, about 60% of Sweden’s population were vaccinated against swine flu [14,15]. In 2010 came the first reports from Finland and Sweden of narcolepsy among children and adolescents vaccinated with one of the vaccination types, Pandemrix. Since then, studies from Sweden, Finland, the United Kingdom, and Ireland have demonstrated a link between narcolepsy and the Pandemrix vaccine, with this vaccine producing a 3-fold increase in the risk of narcolepsy [1]. When the link between the vaccine and narcolepsy was clarified, claims were made by the families of the affected children, and later also by the adolescents themselves, for compensation from pharmaceutical companies, medical insurers, health authorities, and others. In Sweden, health care, including disease prevention, is regulated and performed by the public sector; consequently, the government was the main target of these demands. Among other things, discussions revolved around the maximum limit of compensation. Table 1 lists the most significant events during the period from March 2009, when the virus was detected, to July 2016, when the dispute in Sweden was settled by legislation regulating compensation to affected individuals. Different actions to call for higher compensation were initiated, using channels such as newspapers, television, and the internet.

**Table 1:** Significant events concerning the swine flu outbreak, vaccination, narcolepsy, and compensation regulation.

Date	Event
March 2009	Reports emerge of a new type of swine flu in California and Texas and spreading to Mexico.
June 2009	The World Health Organization declares a swine flu pandemic. Vaccine development begins.
October 2009	The Swedish vaccination campaign starts.
August 2010	Reports of narcolepsy in vaccinated children emerge from Finland and Sweden.
July 2011	The European Medicines Agency recommends limited use of Pandemrix in people <20 years of age.
October 2011	The first persons who had narcolepsy after vaccination receive compensation from the Swedish pharmaceutical insurance industry.
March 2013	The first register study is published, showing the link between Pandemrix and narcolepsy, as also exposed in the daily media.
May 2015	The Swedish government sets the maximum compensation amount for lost income at SEK 10 million.
July 2016	A new law is passed regarding compensation from the Swedish pharmaceutical insurance industry to affected persons who experienced the first symptoms of narcolepsy <24 months after vaccination.

### Objective

The aim of this study was to examine how information was spread, and how opinions and beliefs about narcolepsy as a consequence of Pandemrix vaccination were formed through discussions on social media. For this purpose, we examined a series of messages posted on social media, as well as connections among people participating in the discussions. One research question was whether, in general, social media groups for persons affected by narcolepsy were factual and constructive and helped those with narcolepsy to cope with the situation, and whether the negative campaigning against vaccination came from other sources. If this were the case, then social media were not the main driver of nonfactual discussions; rather, group composition was.

This study was part of a project investigating the life situations of narcolepsy-affected young persons, and the use and meaning of social media and the internet as a means of support and communication of opinions [2]. We hoped that the results of the study would increase knowledge of how the use of social media can affect trust in health care and attitudes toward future health campaigns, and thereby assist in developing interventions to support increased trust in and compliance with such campaigns.

## Methods

### Identification of Discussion Forums

This study was a descriptive retrospective analysis of a series of messages posted on social media, as well as connections between users, between September 2010 and July 2016. We conducted the study in 2017. We found 18 Swedish discussion forums on the Web that concerned narcolepsy and that had started around 2010. As Table 2 shows, in most of these, the volume of activity was very low, with only a few posts. Most of the forums also had no posts from recent years: the Pandemrix vaccination took place in 2010, the first cases of narcolepsy occurred the same year, and most posts in these groups were from around that time. As we were interested in online discussions, where people can meet, take part on equal terms, and go in-depth into issues they find important, some of the sites in Table 2 were irrelevant. Numbers 15 to 18 were blogs, which means they were not really discussion forums. Even though some blogs allow comments, only the blog owner can make posts. Number 17, YouTube, also is not a discussion forum but a publication site. We also considered Twitter (number 11) to be irrelevant because Twitter is by its format not suitable for lengthy discussions, and possibly for that reason, there were no discussions, only a few disconnected tweets.

We selected the only group that had a large number of threads and where activity had been high throughout the 6 years—on average 23 posts per month (number 1). This is a Facebook group in Sweden named “Narcolepsy after the Pandemrix Vaccine,” which had 774 members as of September 12, 2016.

The second most active group was Flashback with 250 threads, or about 3 per month on average. We considered that a quite low activity, in particular because many discussions were offensive, promoted conspiracy theories, or were not about narcolepsy but about entirely different topics, such as fluoride-free toothpaste. Textbox 1 shows some examples (translated from the Swedish language).

The Facebook group is presented as “a group for us, or relatives, who have the diagnosis of narcolepsy or have similar symptoms like narcolepsy/sleeping sickness after vaccination.” Although the group is open, it is reasonable to assume that most people in the group are either affected or close relatives of those affected by the disorder. When the group started, a total of 200 people were affected by, that is, had a diagnosis of, narcolepsy after vaccination. Adding a few who had the symptoms but did not yet have a diagnosis, and between 2 and 3 relatives per affected person, brings the total to around 774. This suggests that the Facebook group was (voluntarily) limited to narcolepsy patients and their close relatives. The group has been active since September 2010, and at the time of our investigation there were 1671 posts, which had generated a total of 10,906 comments.

**Table 2:** Forums found by searching for “narcolepsy,” September 12, 2016.

Forum		Web address	Number of threads up to September 12, 2016	Activity
1	Narcolepsy after the Pandemrix vaccine	https://www.facebook.com/groups/122068704510686/	1671	High and regular
2	Flashback	https://www.flashback.org/sok/Narkolepsi	250	Sporadic and not always about narcolepsy
3	Fragbite	http://fragbite.se/search/?q=Narkolepsi&t=forum	3	Sporadic
4	Allt för föräldrar	http://www.alltforforaldrar.se/snack2/search.php?searchid=357008	59	Low
5	NeuroFörbundet	http://neuroforbundet.se/l.aspx?u=http://neuroforbundet.se/community/grupper/forum/?clubId%3d8	Requires login	N/A^a^
6	Hamsterpaj	http://www.hamsterpaj.net/soek/#!/000100/narkolepsi	1	Low
7	Vetenskap och folkbildning	http://forum.vof.se/index.php	N/A	N/A
8	MEF	http://me-cfs.se/mef-forum/index.php?topic=662.msg3466#msg3466	1	Low
9	Netdoktor.se	http://www.netdoktor.se/neurologi/diskussioner/	1	Low
10	Fuska.se	http://fuska.se/forum/index.php?app=core&module=search&do=search&fromMainBar=1	4	Sporadic
11	Twitter (#Narkolepsi)	https://twitter.com/hashtag/Narkolepsi https://twitter.com/search?q=Narkolepsi&src=typd	No discussions were found, only sporadic unconnected tweets	N/A
12	Narkolepsiföreningen (Facebook)	https://www.facebook.com/narkolepsiforeningen/	N/A	Low
13	Doktorn.com (Facebook)	https://www.facebook.com/doktornpunktcom/	N/A	Low
14	YouTube	https://www.youtube.com/watch?v=d7HGfPrtlkY	N/A	Low
15	Tankebrott blogg	https://tankebrott.nu/2010/08/24/orsak-verkan-narkolepsi-och-pandemrix-och-frustration/	N/A	High
16	Matildas blogg	http://dagsattvakna.for.me/narkolepsi.html	N/A	Low
17	Rune Lanestrands blogg	https://runelanestrand.wordpress.com/tag/narkolepsi/	N/A	Low
18	Svensson blogg	http://blog.zaramis.se/tag/narkolepsi/	N/A	Low

^a^N/A: not available.

**Examples of threads on Flashback.**

- “A friend told [me] that vaccines that we get as kids make us weird, like gay and other stuff.”
- “I read that some kids get autism and other shit.”
- “If you can get narcolepsy from vaccine you can in principle imagine that you can for example become gay.”
- “Vaccines generally give more diseases. More and more people begin to understand that vaccination has become one of the most profitable activities for the pharmaceutic companies. There are many indications that they manipulate statistics, and when you look at it the way you should, vaccines have no or very poor effect. All statistics I have seen suggest that.”
- “‘BLOODY CREEPS.’ I am fucking boiling! How the hell can they have the stomach to complain about that [government compensation to people who got narcolepsy from a vaccine]. They can retire and not do any work for the rest of their lives. I fucking think that those who complain should lose all their compensation!”

### Data Collection

We collected some data directly from the Facebook group, such as a count of posts (a “post” is the start of a discussion on Facebook) and comments. For the content analysis, we selected posts with more than 10 comments from months with high activity, defined as 200 or more posts or comments per month. This selection criterion yielded 141 posts out of the total of 1671 that were available at the time. The reason for this selection was that we wanted to see what the discussions that had “caught on” were about. Posts that generated many comments could be considered to have been more interesting to the group than those that do not. While the limit of 10 comments was somewhat arbitrary, it weeded out posts that received only marginal comments (eg, “good to hear from you”), corrections (eg, “sorry, 25 is the correct number”), or clarifications (eg, “Where did you hear that?” “In...;” “But they made an update this morning saying...”). The selection includes 141 of the 1671 (8.4%) total number of posts and 3086 of 10,906 (28.3%) comments. The remaining 1530 posts (that we did not select) had an average of 5 comments.

We downloaded text data from the Facebook group using Netvizz v1.31 [16] to identify posts with more than 10 comments. We used NVivo 11 for Mac (QSR International) for quantification of data (ie, analyzing the numbers of posts and users).

### Data Analysis

Text data were sorted by the third author (RB) into posts and comments regarding users. We used an inductive approach, which involved reading the data and identifying headings describing the content of the posts and comments. The analysis was conducted in several iterations involving all authors, and including discussion of interpretations of what was said in posts and comments, and various alternatives for quantifying the results, for example by author, keywords, and frequency. The analysis process was driven by a set of analytical questions regarding content, participation, and communication style. The content questions were (1) What are the most common discussion topics? (2) How do discussion topics change over time? and (3) What triggers the major discussions (eg, external events such as news media stories or government or health care system actions, or internal events such as somebody working to raise awareness or personal experiences shared)? The participation questions were (1) How many people or how large a share of group members participate in discussions? and (2) How many people *start* discussions? The question pertaining to communication style was What is the general tone of the communication (positive—negative, factual—polemic, personal—general)?

### Ethical Considerations

As mentioned above, the Facebook group is open and all posts and comments, as well as the names or pseudonyms of the people who have written them, are publicly visible. In this study, no individual’s post or comment in the group could be identified, either by group member name or by the content of their posts. We handled data in line with the Declaration of Helsinki [17]. According to Swedish law, studies on social media do not require ethical approval; however, the overall project was approved by the Regional Ethical Review Board of Uppsala (registration no. 2013/505).

## Results

### Content

**Figure 1:**
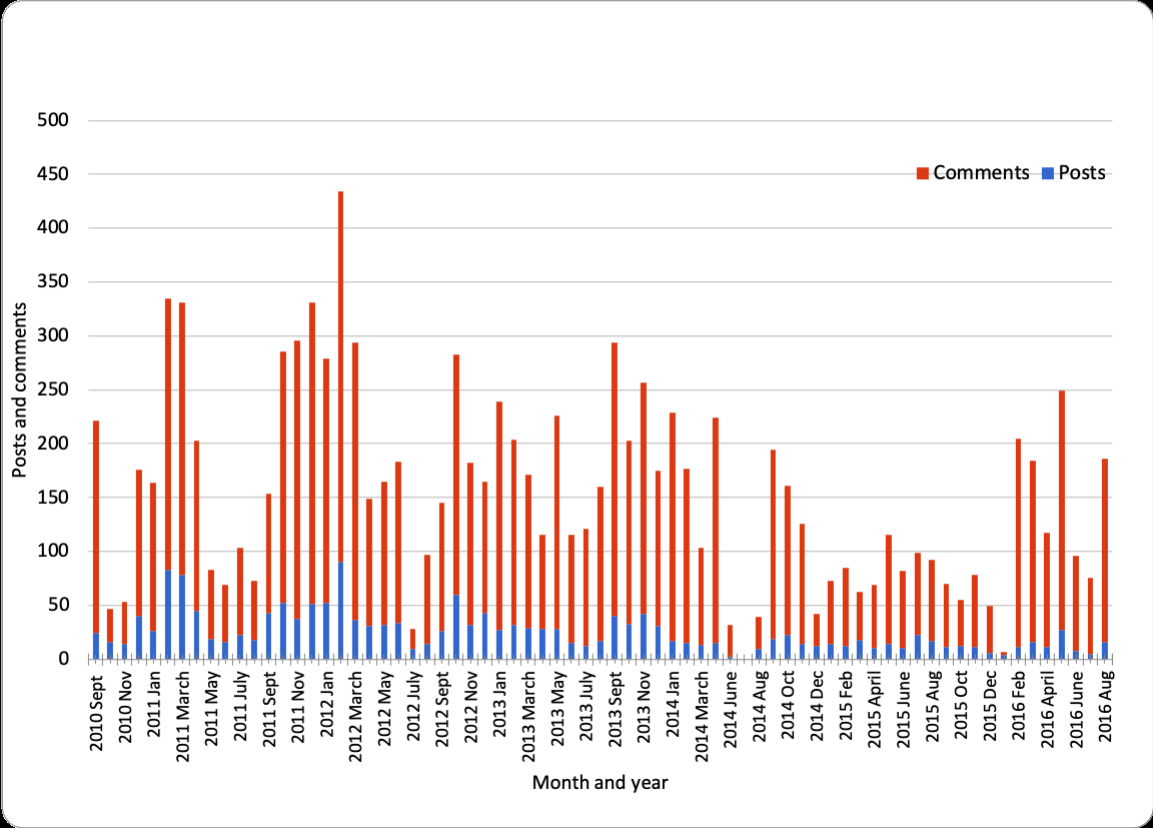
Posts and comments over time.

The Facebook group activity was fairly high throughout the years 2010 to 2016, although there was considerable monthly variation (Figure 1). The peaks of activity corresponded to the major events over the years, that is, the first cases of narcolepsy in late 2010, the first compensation paid in late 2011, and the passage of a law on compensation in July 2016 (see Table 1). But there was also sustained high activity from late 2012 to early 2014. While no milestone event took place during that period, it was a time when new cases of narcolepsy were being discovered and many more people found themselves struggling with a difficult situation.

**Table 3:** The most common discussion topics.

Rank	Topic	Number of posts
1	Adverse effects of Pandemrix or the vaccination	37
2	Drugs and medication	28
3	Narcolepsy diagnosis	17
4	Insurance and compensation	16
5	Doctor or hospital visits and treatments	12
6	Vaccination in general	8
7	Events and meetings	8
8	Upset discussions about public statements by an official or journalist	5^a^
9	Scientific research	2
10	Other	8^b^

^a^This includes statements by radio hosts (n=2), politicians (n=2), and the Head of the Swedish Institute for Infectious Disease Control (n=1).

^b^These include 8 different topics with only 1 post each, ranging from Christmas greetings to blood donation to a conspiracy theory about whether the government had known about the adverse effects.

### Changes in Discussion Topics Over Time

Table 3 lists the most common discussion topics.

**Figure 2:**
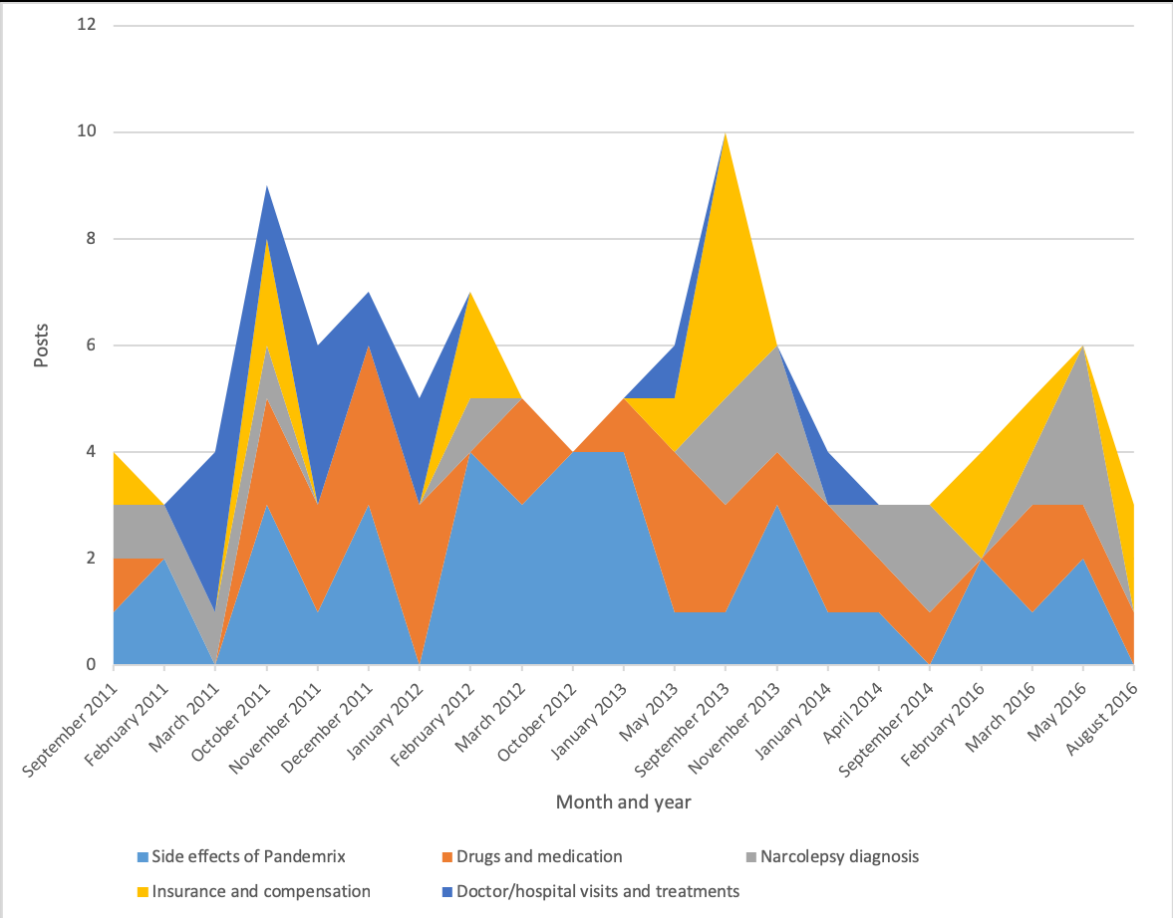
Post topics with high activity.

**Figure 3:**
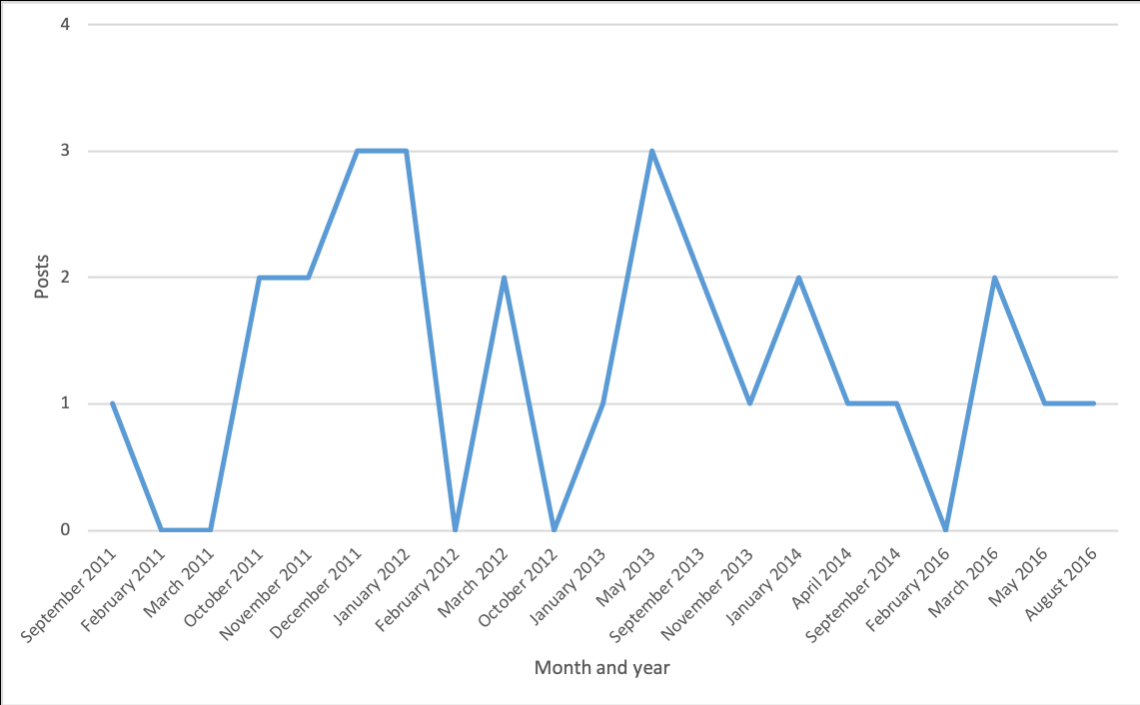
Posts on drugs and medication.

**Figure 4:**
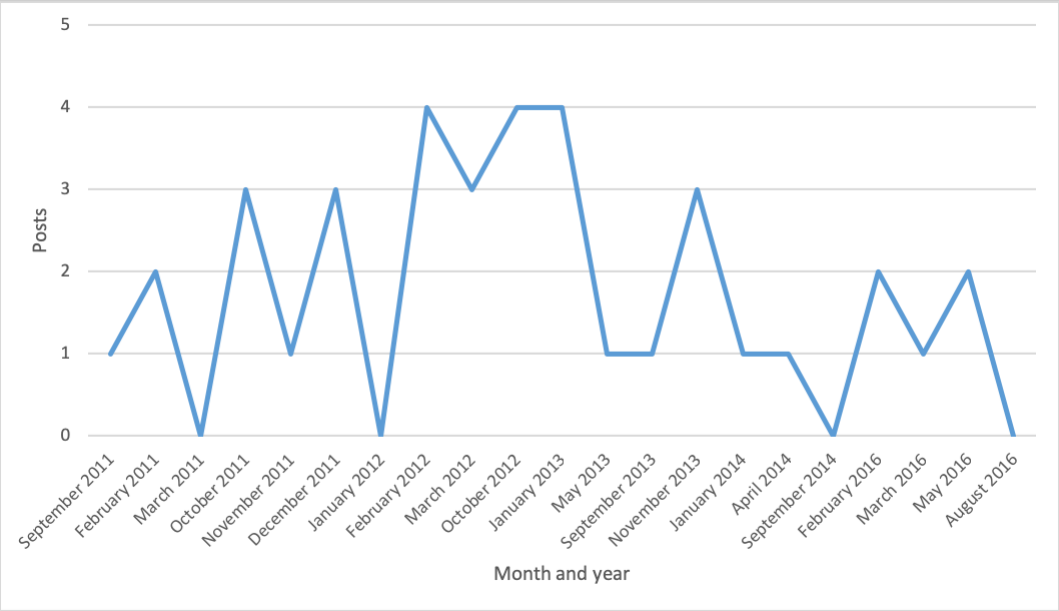
Posts about the adverse effects of Pandemrix.

**Figure 5:**
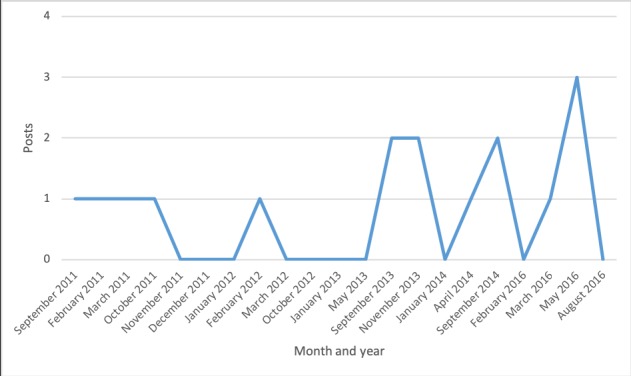
Posts about narcolepsy diagnosis.

**Figure 6:**
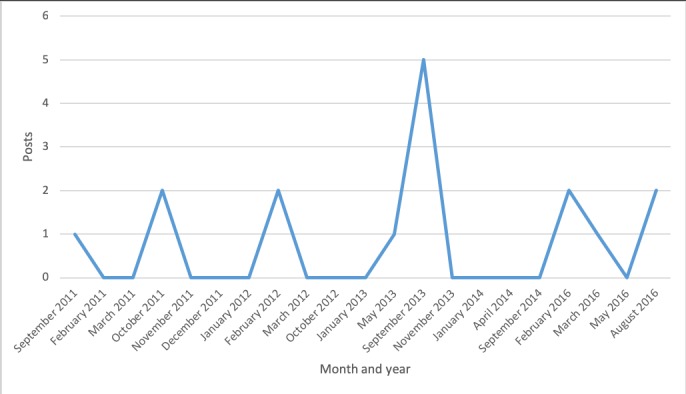
Posts about insurance and compensation.

**Figure 7:**
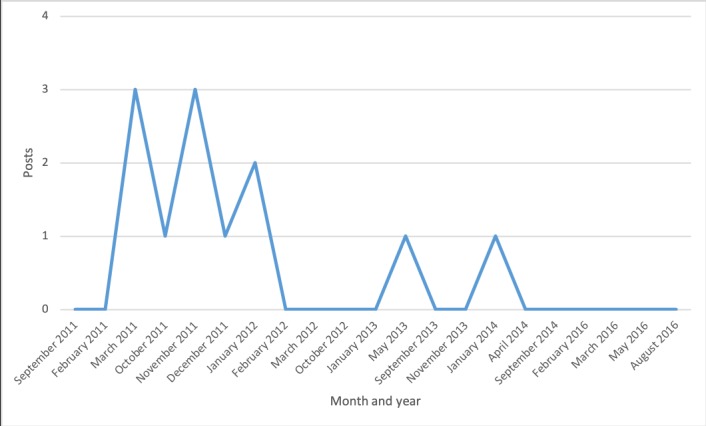
Posts about doctor or hospital visits and treatments.

Discussion topics changed over time (Figures 2-7). Some topics, such drugs and medication (Figure 3) or narcolepsy diagnosis (Figure 4), were on the agenda more or less all the time, albeit with varying frequency. Peaks corresponded to significant narcolepsy events, such as, initially, the increase in cases in 2011–2012, then the corroborated link between the vaccination and the disease in 2013. Adverse effects also remained a hot topic for a long time, peaking in late 2012 and 2013 when the relation to the vaccination was first investigated and later established (Figure 4). Other themes, such as insurance and compensation (Figure 6) and doctor or hospital visits and treatments (Figure 7), emerged frequently for shorter periods, following significant events after the vaccination campaign had started in 2010 (see Table 1). Doctor or hospital visits was a frequent topic during 2011–2012 when the pandemic exploded. It was followed by another, smaller peak in May 2013, which coincided with, and could be related to, increased publicity at the time about the link between Pandemrix and narcolepsy, based on the first Swedish registry study [14,15] published on the website of the Swedish Medical Products Agency in 2013 and exposed in the daily media. The topic was then related to the importance of having a diagnosis in order to qualify for compensation. The topic narcolepsy diagnosis was a frequent discussion topic from 2013, when the cause was established and discussions on compensation became active in the media, until 2016, when the new law settled the compensation issue. Insurance was another major topic in late 2013, when the first study established the link between Pandemrix and narcolepsy, which naturally triggered discussion about compensation.

Even the more constant topics had ups and downs over the investigated period. For example, discussions on the adverse effects of Pandemrix were frequent throughout the period, but there was a major peak from February 2012 to January 2013, coinciding with discussion in the media during that period (Figure 4).

**Table 4:** Rationale behind the posts.

Rationale	Number of posts
Searching for information or others’ experience	46
Sharing personal experience or information	44
Sharing information from external sites	27
Sharing information about an upcoming event or meeting	8
Airing statements by the media	8
Other	7
Presenting a conspiracy theory	1
Total	141

### Discussion Triggers

While peaks in discussion topics can be related to significant events in the history of the vaccination and the ensuing pandemic, there is still the question of which events, situations, needs, or emotions triggered specific major discussions. As Table 4 shows, the most common cause by far prompting the writing of posts was a personal need for finding and sharing information. Only a small number of posts (n=8) contained “negative” discussions. These were reactions to public statements by officials or journalists that were felt to be derogatory. Only 1 post aired a conspiracy theory, and this did not take root.

The general tone of the discussions was factual, friendly, and positive despite the problematic, to say the least, situation many of the group members found themselves in. They tried to share information and experiences with each other in the hope of easing the life of “the victims.” The posts were generally by a member seeking answers and experiences from the other members of the group, or someone wanting to share information or personal experiences with the group.

Of the 141 posts, only 13 (9.2%) had a more negative character, and even in these threads the tone was mainly factual. When the members expressed anger in their posts, it was anger they felt either toward individuals (politicians, officials, or a media host) who had made statements about the situation that group members felt were unfair or derogatory (n=4) or toward individuals who had purportedly exploited the situation to commit insurance fraud (n=1), or the posts expressed dissatisfaction with a doctor (n=2) or anger toward the Head of the Swedish Public Health Agency (n=1). Alternatively, it was about insurance and compensation (n=2), about incorrect information on a website (n=1), or demands for a public apology from the government (n=1). In one case a conspiracy theory claiming that the government had known about the adverse effects beforehand was posted.

### Participation

The Facebook group had 774 members by the time of our investigation. Over the group’s lifetime to that point, a total of 1671 posts had been published, with 10,906 comments. We selected the major discussions: those that generated 10 or more comments.

In all, 59.7% (462/774) of the group members had participated in the discussions by either creating or commenting on a post. Slightly more than one-third of the group members (277/774, 35.8%) had created a post (that is, started a discussion).

The 5 people who contributed the largest number of discussions accounted for 26.0% (435/1671) of the posts published in the group. They also provided 22.2% (2423/10,906) of all the comments on the forum.

The 10 people (10/774, 1.3% of the population) who created the largest number of discussions accounted for 42.19% (705/1671) of the posts that generated the most comments. They also provided 35.19% (3838/10,906) of all comments on the forum. The single most active member had created 107 posts, or 6.4% of the total 1671 posts. A total of 7 members had created 50 or more posts each, and 32 members had created more than 10 posts. All the other 445 active posters had provided an average of 1.7 posts.

These participation rates are considerably higher than the general rule of thumb for social media use. The “90%-9%-1% rule” [18] states that, in most online communities, 1% of users account for almost all the action, 9% of users contribute a little, and 90% of users are lurkers who never contribute. In the community we analyzed, we found agreement on the last statement of the rule but not the first or second. In this population, 1.2% (9/774) of participants accounted for 41.8% (281/671) of the posts and 35.5% (3871/10,906) of the comments. While not statistically constituting the majority, this is certainly a major part. We also found that the majority of members of the community—59.0% (457/774) if we take away the most active 1%—contributed at least something. The share of lurkers, if defined as people who never actively participate, in our study was then only 40.1% (310/774). Even though many had made only 1 or a very few posts, posting means starting, or trying to start, a discussion, which can be considered more active participation than commenting on someone else’s post.

Generally speaking, the Facebook group we studied can be considered fairly participatory by general social media standards, even though a few people clearly dominated the discussions. Presumably this means not only that participants considered the group to be highly relevant, but also that active participation was important, and that many people trusted the group enough to share even sensitive stories from their lives.

## Discussion

### Principal Findings

The aim of this study was to examine how information was disseminated, and how opinions and beliefs about narcolepsy as a consequence of Pandemrix vaccination were formed by discussions on social media. To investigate this, we examined a series of messages posted on social media over a 6-year period. The focus was on people affected by narcolepsy after having been vaccinated against swine flu. Out of 18 Swedish groups originally discussing this topic, the Facebook group we examined was the only one that has remained active throughout the years.

Our research question, whether social media groups for affected patients are generally factual and constructive and help the patients cope with the situation and, further, whether negative campaigning against vaccination comes from other sources, was motivated by a more general research interest. We assumed that social media do not inherently drive nonfactual discussions or less honest discussion styles but, rather, that social media are a useful tool not just for social purposes but also for really difficult discussion on very serious matters that concern people’s health. If this assumption holds, then social media can be very useful not just for the people participating but also for health care providers. Interventions using social media, such as Facebook, in health care are increasing—for example, interventions for awareness of breast cancer [19], physical activity promotion among adolescent and young adult childhood cancer survivors [20,21], sexual health promotion [22], HIV communication [23], and enhancement of positive health outcomes among adolescent and young adults [24]: all studies referenced here indicated positive benefits. However, there is still a lack of robust evidence of effects and concerning how to best design such interventions. Despite an increased use of social media by health care providers, issues remain concerning how to best provide health information and support that are trustful and that promote healthy behavior among people. The Facebook group studied here was self-organized, and this may have been a factor affecting people’s trust in it.

If social media themselves do not drive nonfactualism and asocial behavior, then certainly people and group composition might. This particular group was composed of people who shared the same serious situation and great need, not just for information, but also for advice and the possibility to share their concerns with others. It should be noted, however, that most of them did not know each other in real (offline) life. They lived in different cities and were dispersed across Sweden. In this respect theirs was a truly virtual community.

From previous research on social media we know that distance makes social ties weaker. Harsh language is more common when people live far apart from each other than when they live in the same city and there is at least a theoretical chance that they might physically meet. It appears that in the case we studied, the shared situation served the purpose of making people feel close enough to make the community “real.” This was supported, for example, in studies of a group for persons with Huntington disease, where exchanging informational and emotional support was a key function [25], and a group for persons with amyotrophic lateral sclerosis that served as a source for distributed knowledge [26].

This type of scenario creates opportunities for health care, as many patients with some serious condition share the same situation: they need to learn about their problem and possible treatments, and they have to cope with their situation. They often do not know people with the same condition at the outset but want to find them as the need arises. Whatever the condition, in most cases most people with the same condition do not live next door. Some people prefer to share and disclose experiences of illness and health in forums such as Facebook, while others are hindered by the lack of anonymity in social media [27]. The resource we presented might also be limited to people with internet skills and access, and may exclude people from socially disadvantaged groups with lower socioeconomic status [28].

Of course, 1 case does not prove a hypothesis, but at least this case gives positive evidence of the possibilities that social media can present to these people living with an uncommon but serious condition in a difficult and uncertain situation. Despite several years having passed since the onset of the first vaccination-related cases of narcolepsy, this Facebook group still exists. This could indicate a need for support—support from society and, even more important, from health care services.

Although the discussions in the group were generally factual, positive, and directed toward problem solving and coping, rather than bitterness and conflict, we did find one post putting forward a conspiracy theory. While there may have been more negativity in the posts with fewer than 10 comments, these did not lead to any longer discussion. Much has been written about how social media amplifies opinions [6-8], but it has also been found that conflict in discussion forums often occurs between groups rather than within them. For example, at “hate sites,” traditional media are criticized while criticism against hate sites mainly occurs at traditional media sites. The same seems to be the case here. There was little conflict in this group, either internally or externally (eg, through members attacking people outside the group), but it is easy to find other places on the Web where antivaccination views are vigorously and often aggressively expressed. A systematic review of 42 studies, 16 of which explored Facebook posts, concluded that most had a beneficial or neutral impact on the clinical outcome of chronic diseases [29].

It would be interesting to investigate why some posts did not generate comments—was it because they were inappropriate, marginal, or made by people who were not centrally positioned in the group? This investigation, however, was not about the social situation of the group per se, but rather about the general role of a group such as this, regarding discussions about a severe disorder.

### Conclusion and Implications

The aim of this study was to examine how information was shared, and opinions and beliefs about narcolepsy as a consequence of Pandemrix vaccination were formed by discussions on social media. To investigate this, we examined a series of messages posted on social media over a 6-year period. We found, first, high group activity throughout the years 2010 to 2016, with peaks corresponding in time to major narcolepsy-related events, such as the appearance of the first cases in 2010, the first compensation paid in late 2011, and passage of a law on compensation in July 2016. Second, unusually, a majority (about 60%) of the group members took part in discussions and only 40% were lurkers (in contradiction to the 99%-9%-1% rule of thumb for participation in an online community). Third, the conversation in the group was largely factual and had a civil tone, even though there was a long period of struggle to get acknowledgement of the link between the vaccine and the disease and regarding the compensation issue. Fourth, radical, nonscientific views, such as those of the antivaccination movement, did not shape the discussions in the group but were active elsewhere on the internet.

The Facebook group we studied is a good example of social media use for patient self-help in a difficult situation. The example shows that social media do not by themselves induce trench warfare but, given a good group composition, can provide a necessary forum for managing an emergency where health care and government have failed and are mistrusted, and patients have to organize themselves so as to cope.

The critical factor is not social media use, but group composition. The Facebook group studied here appeared to have consisted only of people directly concerned—individuals directly affected and their close families, and, furthermore, people who appeared to believe in facts and science, even though the pandemic was started by a health care mistake. This socially coherent and fact-oriented discussion group survived 6 years, while 17 other groups, many of which included much more confrontational language and views, that appeared at the outset of the crisis soon faded away.

This means that trust in government and health care is very important for the outcome of social media discussions. It must be strong enough to survive even tough challenges, such as this 6-year-long struggle. This trust cannot exist only beforehand but must be reinforced during the process.
